# Acoustic structure quantification by using ultrasound Nakagami imaging for assessing liver fibrosis

**DOI:** 10.1038/srep33075

**Published:** 2016-09-08

**Authors:** Po-Hsiang Tsui, Ming-Chih Ho, Dar-In Tai, Ying-Hsiu Lin, Chiao-Yin Wang, Hsiang-Yang Ma

**Affiliations:** 1 Department of Medical Imaging and Radiological Sciences, College of Medicine, Chang Gung University, Taoyuan, Taiwan; 2 Institute for Radiological Research, Chang Gung University and Chang Gung Memorial Hospital at Linkou, Taoyuan, Taiwan; 3 Department of Medical Imaging and Intervention, Chang Gung Memorial Hospital at Linkou, Taoyuan, Taiwan; 4 Department of Surgery, National Taiwan University Hospital and College of Medicine, National Taiwan University, Taipei, Taiwan; 5 Department of Gastroenterology and Hepatology, Chang Gung Memorial Hospital at Linkou, Chang Gung University, Taoyuan, Taiwan; 6 Graduate Institute of Clinical Medical Sciences, College of Medicine, Chang Gung University, Taoyuan, Taiwan

## Abstract

Acoustic structure quantification (ASQ) is a recently developed technique widely used for detecting liver fibrosis. Ultrasound Nakagami parametric imaging based on the Nakagami distribution has been widely used to model echo amplitude distribution for tissue characterization. We explored the feasibility of using ultrasound Nakagami imaging as a model-based ASQ technique for assessing liver fibrosis. Standard ultrasound examinations were performed on 19 healthy volunteers and 91 patients with chronic hepatitis B and C (*n* = 110). Liver biopsy and ultrasound Nakagami imaging analysis were conducted to compare the METAVIR score and Nakagami parameter. The diagnostic value of ultrasound Nakagami imaging was evaluated using receiver operating characteristic (ROC) curves. The Nakagami parameter obtained through ultrasound Nakagami imaging decreased with an increase in the METAVIR score (*p* < 0.0001), representing an increase in the extent of pre-Rayleigh statistics for echo amplitude distribution. The area under the ROC curve (AUROC) was 0.88 for the diagnosis of any degree of fibrosis (≥F1), whereas it was 0.84, 0.69, and 0.67 for ≥F2, ≥F3, and ≥F4, respectively. Ultrasound Nakagami imaging is a model-based ASQ technique that can be beneficial for the clinical diagnosis of early liver fibrosis.

The major consequence of chronic liver diseases is the increased deposition of fibrotic tissues within the liver, resulting in the development of cirrhosis and hepatocellular carcinoma^1^. Percutaneous liver biopsy is currently the gold standard for assessing liver fibrosis. Because of the invasiveness of and sampling errors^2^,^3^ in liver biopsy, many researchers have been focusing on developing imaging techniques for the noninvasive evaluation of liver fibrosis. Among all possibilities, ultrasound provides real-time screening of the liver and has been widely used in routine clinical examinations. The most commonly used ultrasound methods for assessing liver fibrosis are ultrasound elastography techniques, such as transient elastography, acoustic radiation force impulse imaging, and shear wave elastography^4^. Although liver stiffness is strongly correlated with the fibrosis stage, elastography only assesses stiffness and not fibrosis^5^. In all histological fibrosis scoring systems, the stages are based on morphological analyses, wherein the formation of septa and nodules is the major factor^6^. Therefore, morphological analysis–based conventional grayscale ultrasound may contain valuable information beyond that provided by elastography for assessing liver fibrosis.

The acoustic structure quantification (ASQ) technique has recently gained attention as a tool for characterizing liver parenchyma^5^,^7^,^8^,^9^,^10^. ASQ is performed on a basic pulse-echo ultrasound grayscale system for characterizing tissues through the statistical analysis of the acquired ultrasound echo signals^11^. Because normal liver parenchyma consists of a three-dimensional arrangement of microstructures that are smaller than the wavelength of ultrasound used clinically (unresolvable scatterers), the statistics of the echo amplitude follow Rayleigh distribution^11^,^12^,^13^,^14^. During liver fibrosis, the fibrotic structures and nodules develop and become larger than the wavelength (resolvable scatterers), resulting in a relatively high degree of variance in the scattering cross-sections of scatterers. In this condition, the echo texture is overall coarse and heterogeneous, and the echo amplitude distribution deviates from the Rayleigh distribution. Using ASQ, the degree of deviation from the Rayleigh distribution can be quantified for assessing liver fibrosis. The ASQ software has been commercialized and is available in Toshiba ultrasound scanners (Apilo XG, Toshiba Medical Systems, Otawara, Japan).

The echo amplitude statistics can be quantified more precisely by estimating the shape parameters in the statistical models. The Nakagami parameter (denoted by *m*) of the Nakagami distribution is a simple and general method for describing all conditions of the echo amplitude distribution^15^,^16^,^17^,^18^. Numerous studies have revealed that Nakagami parameter–based ultrasound parametric imaging can visualize changes in the echo amplitude distribution; therefore, it has already been used in various applications, such as breast tumor classification^19^, cataract detection^20^, vascular flow analysis^21^, thermal ablation monitoring^22^, heart muscle characterization^23^, and liver fibrosis evaluation in rats^24^,^25^. Previously, we preliminarily validated the feasibility of ultrasound Nakagami imaging for detecting liver fibrosis in humans. It was shown that the fibrosis score based on the features of liver surface (smooth, irregular, or undulated), liver parenchyma (homogeneous, heterogeneous, or coarse), hepatic vessel (smooth, obscure, or narrow), and spleen size (< 20 cm^2^ or >20 cm^2^) correlates with the severity of liver fibrosis^26^, and we observed that the Nakagami parameter correlated with the sonographic fibrosis score assigned according to the above features of the ultrasound image^14^.

To further investigate the diagnostic ability of Nakagami imaging for assessing liver fibrosis, histological findings should be used as the ground truth for performance evaluations. This study investigated the relationship between the Nakagami parameter and histological findings in patients with liver fibrosis.

## Materials and Methods

### Study Population

This prospective study was approved by the Institutional Review Boards of National Taiwan University Hospital and Chang Gung Memorial Hospital. All participants signed informed consent forms. All the experimental methods were carried out in accordance with the approved guidelines. Between August 2014 and July 2015, we enrolled 19 healthy volunteers from Chang Gung University as the control group and 91 patients (59 patients from Taiwan University Hospital and 32 patients from Chang Gung Memorial Hospital) as the study group. The volunteers had no drinking and smoking habits, no remarkable past medical history, and no clinical symptoms and signs of liver and renal parenchymal diseases. Patients with confirmed chronic hepatitis B or C infection scheduled for liver biopsy or partial liver resection were recruited.

### Clinical and laboratory examination

Age, sex, weight, height, and body mass index (BMI) was recorded for each patient. Venous blood samples collected after overnight fasting for 8 h were used for measuring the aspartate aminotransferase (AST) level, platelet (PLT) count, and AST-to-PLT ratio (APRI).

### Ultrasound Examination

A portable clinical ultrasound scanner (Model 3000, Terason, Burlington, MA, USA) and a convex transducer with a central frequency of 3 MHz, 128 elements, and a pulse length of approximately 2.3 mm (Model 5C2A, Terason) were used for sonographic imaging. All participants underwent a standard-care ultrasound examination. For each participant, five valid grayscale images of the liver parenchyma (no acoustic shadowing artifacts and exclusion of large vessels in the region of analysis) were obtained from the right intercostal view by a gastroenterologist. The focus and depth of imaging were set at 4 and 8 cm, respectively. The grayscale image data were stored as a ULT file dedicated to the Terason ultrasound system. Raw echo signal data consisting of 128 scan lines corresponding to each grayscale image were obtained by converting the ULT files into MAT files, which were read using the MATLAB software for offline data processing and ultrasound Nakagami imaging.

### Ultrasound Nakagami imaging

Echo amplitude distribution measured from tissues can be classified as Rayleigh, pre-Rayleigh, and post-Rayleigh distributions^27^. The variation of the Nakagami parameter from 0 to 1 corresponds to a change in the echo amplitude distribution from the pre-Rayleigh to Rayleigh distribution; Nakagami parameters higher than 1 indicate that the statistics of the echo amplitude conform to the post-Rayleigh distribution^15^. Therefore, the Nakagami parameter allows the quantification of echo amplitude distributions with specific physical meanings.

The detailed techniques of Nakagami parameter estimation and imaging were described previously^14^,^28^,^29^. In brief, (i) The envelope image was obtained using the absolute value of the Hilbert Transform of each echo signal filtered using empirical mode decomposition^28^; (ii) A square window within the envelope image was used to collect local amplitude data for estimating the Nakagami parameter, which is assigned as the new pixel located in the center of the window. The side length of the window was three times the pulse length of the transducer^14^,^24^,^25^,^27^,^29^. The maximum likelihood estimator (MLE) derived by Greenwood^30^ was selected to calculate the Nakagami parameter because MLE yields a lower variance in estimating the Nakagami parameter^31^,^32^,^33^. (iii) The window was allowed to move throughout the envelope image in a one-pixel step, and step 2 was repeated to construct a Nakagami parameter map, which was further converted into a fan-shaped image according to the geometry of the curve probe. The key factor for a stable estimation of the Nakagami parameter is the axial length of the envelope signal, not the number of data points or the length of the lateral side of the window. As long as the sampling rate of the analog-to-digital converter in the imaging system satisfies the Nyquist theory, the signal waveform can be reconstructed without the aliasing effect to describe the trend of the variation in the envelope amplitude; and (iv) a primary region of interest (ROI) was manually set on the image of the liver parenchyma. The pixel values (i.e., the Nakagami parameter) within the ROI, which served as the biomarker of liver fibrosis, were averaged. The image data processing was performed using MATLAB software (Version R2012a, The MathWorks, Inc., MA, USA).

### Histological analysis

After ultrasound examination, liver resection or percutaneous liver biopsy was performed within 1 wk. For each patient scheduled for liver resection, one specimen taken for histological examination was located far away from the main lesion (>1 cm). For each patient underwent liver biopsy, one specimen was obtained from the right liver lobe through an intercostal approach under ultrasound imaging guidance. All specimens were placed in formalin, and sent to the Department of Pathology for histological examinations. Samples were embedded in paraffin, stained with hematoxylin–eosin (H&E) and picrosirius red (PR), and read on-site by expert liver histologists. Samples showing a minimum of six portal tracts were considered adequate for histological evaluation^5^. Liver fibrosis was semiquantitatively evaluated using the METAVIR scoring system: F0, no fibrosis; F1, portal fibrosis with no septa; F2, portal fibrosis with few septa; F3, bridging fibrosis with many septa; and F4, cirrhosis (nodular stage)^6^.

### Statistical analysis

The Nakagami parameter as a function of the METAVIR score was expressed as the median and interquartile range (IQR). The Pearson correlation coefficient *r* and the probability value (*p*) were calculated for evaluating the correlation between the Nakagami parameter and METAVIR score. Independent *t*-test was performed to compare the difference in the Nakagami parameter between each group (*p* < 0.05 was considered statistically significant). The receiver operating characteristic (ROC) curve analysis at 95% confidence interval (CI) was performed to obtain the area under the ROC (AUROC). The AUROC was used to determine the predictive value of the Nakagami parameter for diagnosing each fibrosis threshold: F0 versus F1–F4 (≥F1), F0–F1 versus F2–F4 (≥F2), F0–F2 versus F3–F4 (≥F3), and F0–F3 versus F4 (≥F4). In addition, sensitivity, specificity, and accuracy were reported. All statistical analyses were performed using SigmaPlot software (Version 12.0, Systat Software, Inc., CA, USA).

## Results

**Figure 1:**
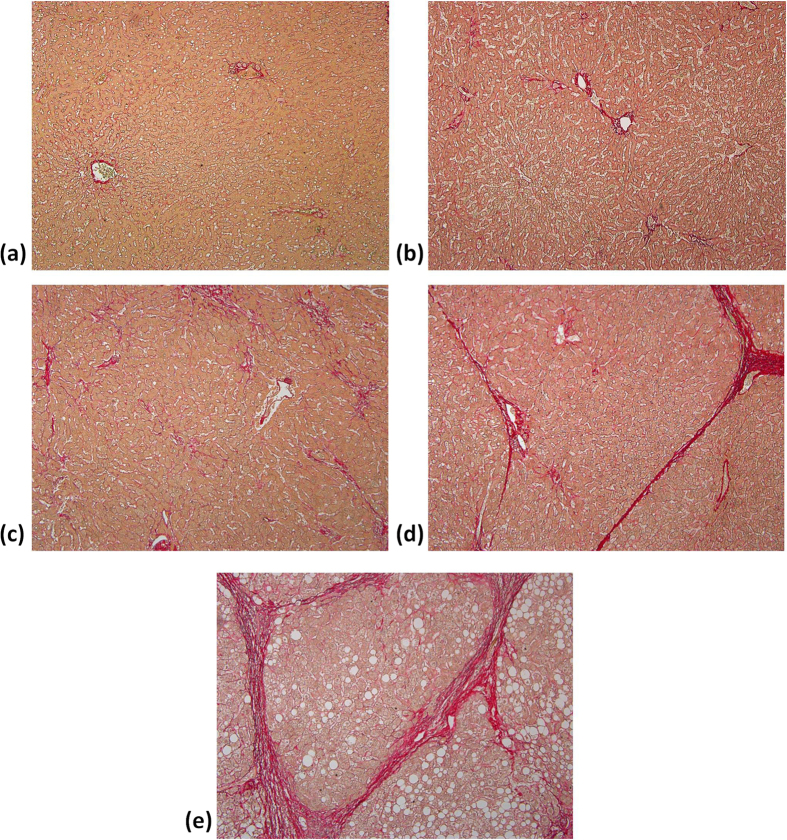
PR-stained sections (100 magnification) obtained from patients’ livers with different degrees of fibrosis from F0 to F4. (**a**) F0: no fibrosis; (**b**) F1: fibrous expansion of portal areas without septa (i.e., portal fibrosis); (**c**) F2: portal fibrosis with few septa was observed; (**d**) F3: fibrous expansion of portal areas with marked bridging or septa (i.e., septal fibrosis); (**e**) F4: the tissue is composed of nodules surrounded completely by fibrosis (i.e., cirrhosis).

**Figure 2:**
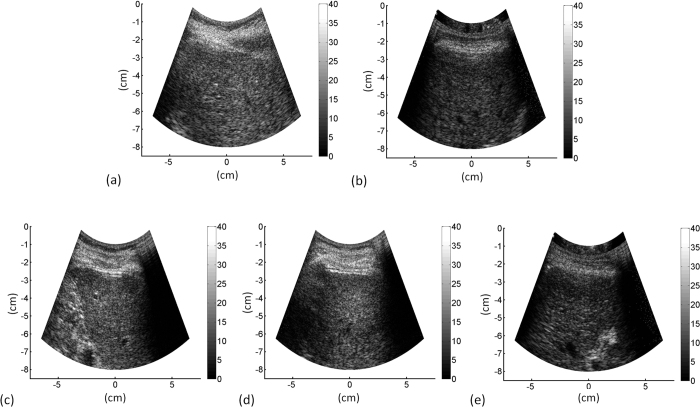
Grayscale B-mode images obtained from the healthy volunteers and patients with liver fibrosis. (**a**) F0; (**b**) F1; (**c**) F2; (**d**) F3; (**e**) F4. B-mode images were reconstructed using log-compressed envelopes of ultrasound signals provided by the Terason ultrasound scanner.

**Figure 3:**
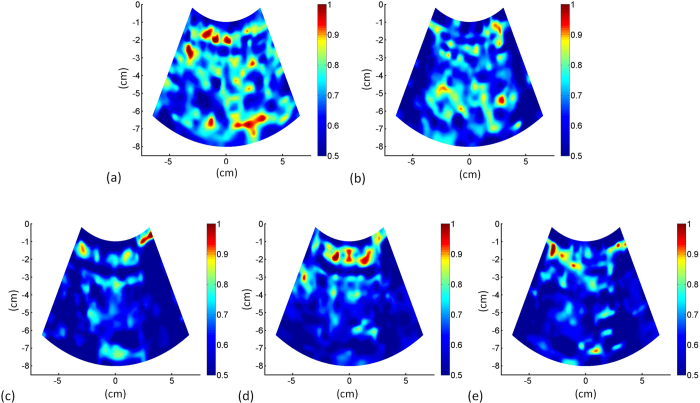
Ultrasound Nakagami images obtained from the healthy volunteers and patients with liver fibrosis. (**a**) F0; (**b**) F1; (**c**) F2; (**d**) F3; (**e**) F4. The brightness of the Nakagami image typically decreased as the METAVIR scores increased from F0 to F4.

**Figure 4:**
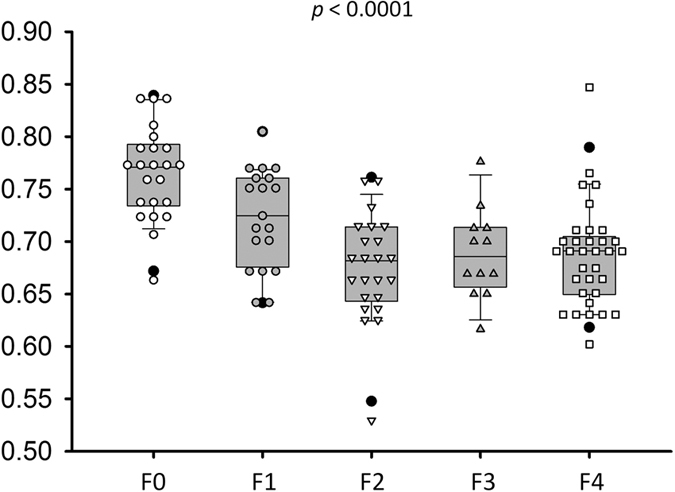
The Nakagami parameters corresponding to each liver fibrosis stage. Data are expressed using box plots. The Nakagami parameter decreased with an increase in the histological fibrosis stage, representing an increase in the degree of pre-Rayleigh statistics for the echo amplitude distribution.

**Figure 5:**
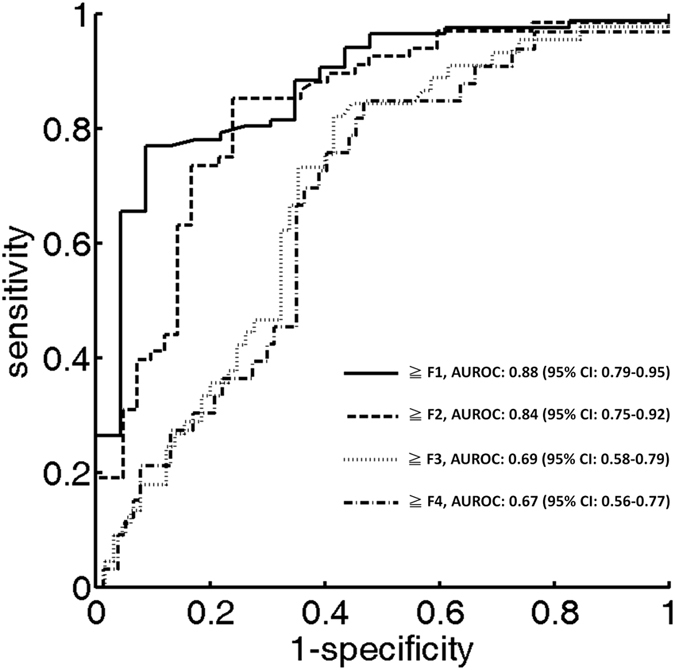
ROC curves for diagnosing different liver fibrosis stages. The AUROC obtained using ultrasound Nakagami imaging in our study was 0.88, 0.84, 0.69, and 0.67 for ≥F1, ≥F2, ≥F3, and ≥F4, respectively.

The characteristics of healthy volunteers in the control group are summarized in Table 1, and the patients’ demographic data and biological and histological findings are summarized in Table 2. Because the volunteers had a normal BMI and no remarkable past medical history or clinical symptoms of liver parenchymal diseases, the control group data were categorized as F0 for comparison with the patient data. The PR-stained section images obtained from patients with different stages of liver fibrosis are shown in Fig. 1. Figures 2 and 3 present the grayscale B-mode and Nakagami images obtained from the healthy volunteers and patients with liver fibrosis, respectively. The brightness of the Nakagami image typically decreased as the METAVIR scores increased from F1 to F4. The Nakagami parameters corresponding to each liver fibrosis stage are presented in Fig. 4. The dynamic range (i.e., the difference between the maximum and minimum values) of the Nakagami parameter was 0.53–0.85. The Nakagami parameter monotonically decreased as the severity of liver fibrosis increased (*r* = −0.45, *p* < 0.0001). The median Nakagami parameter was 0.77 (IQR: 0.73–0.79) for F0, 0.72 (IQR: 0.68–0.75) for F1, 0.68 (IQR: 0.64–0.70) for F2, 0.68 (IQR: 0.66–0.71) for F3, and 0.69 (IQR: 0.65–0.70) for F4. A significant difference was observed between F0 and F1 (*p* = 0.0006) and F1 and F2 (*p* = 0.0033). However, no significant difference was observed between F2 and F3 (*p* = 0.45) and F3 and F4 (*p* = 0.91). The ROC curves for diagnosing different liver fibrosis stages are presented in Fig. 5. The AUROCs (95% CI) were 0.88 (0.79–0.95), 0.84 (0.75–0.92), 0.69 (0.58–0.79), and 0.67 (0.56–0.77) for fibrosis stages ≥F1, ≥F2, ≥F3, and ≥F4, respectively. The performance profile for ultrasound Nakagami imaging is presented in Table 3.

## Discussion

In this study, we proposed ultrasound Nakagami imaging as an alternative for the acoustic structure characterization of liver tissue. A decrease in the brightness of the Nakagami image of the liver represents the liver parenchyma deviating from the normal state (a relatively homogeneous medium) to the fibrotic state (a medium with a relatively high degree of variance in the scattering cross-sections of scatterers). The Nakagami parameter is inversely proportional to the severity of liver fibrosis. The advantage of ultrasound Nakagami imaging is that the echo amplitude distribution can be quantified with a specific physical meaning (i.e., *m* < 1: pre-Rayleigh distribution, *m* = 1: Rayleigh distribution, and *m* > 1: post-Rayleigh distribution). We observed that the Nakagami parameters of normal livers were smaller than 1; this finding is consistent with that of a previous study that reported that the real echo amplitude distribution is not a perfect Rayleigh statistic because of the presence of small vessel walls^10^. As the METAVIR score increased, the Nakagami parameter decreased, representing an increase in the degree of pre-Rayleigh statistics for the echo amplitude distribution.

For clinicians, the most critical question regarding a patient with chronic liver disease is whether the patient has cirrhosis^1^. Ultrasound elastography, including elastography strain imaging, transient elastography, acoustic radiation force impulse imaging, and shear wave elastography, has been demonstrated to produce a reliable evaluation of liver cirrhosis^4^,^34^,^35^. A recently published consensus statement indicated that ultrasound elastography may discriminate between the early (METAVIR scores F0 and F1) and late stages (METAVIR scores F3 and F4) of liver fibrosis^1^. However, hepatic inflammation influences the estimation of liver stiffness^36^,^37^,^38^. By contrast, elastography strain imaging is operator dependent, and both transient elastography and shear wave elastography are performed only when a specific probe is available and system requirements are met. When these functional elastography systems are unavailable in a clinical setting, using a conventional grayscale scanner for yielding liver fibrosis-related information becomes crucial.

Compared with ultrasound elastography, ultrasound Nakagami imaging can be a complementary tool for diagnosing liver fibrosis. Nakagami imaging may be less affected by the inflammatory activity because a study reported no significant correlation between the measurements of the echo amplitude distribution and blood alanine aminotransferase level^5^. Using approaches based on acoustic structure characterization may benefit liver fibrosis assessment in patients with chronic hepatitis B. Moreover, using a morphological ultrasound technique prevents interference from ascites, which is a contraindication for transient elastography^5^. In particular, the algorithms of ultrasound Nakagami imaging are completely compatible with those of a conventional ultrasound B-mode system, making the combination of the routine liver examinations and liver fibrosis assessment more feasible.

The diagnostic value of the conventional ASQ in quantifying the degree of liver fibrosis remains questionable because of inconsistent findings. The performances of ASQ in liver fibrosis assessment reported in literature are summarized in Table 4. Toyoda *et al*.^11^ and Ricci *et al*.^39^ have shown that ASQ can differentiate between individual fibrosis stages. No significant correlation was observed between ASQ parameters and liver fibrosis stages in Kramer *et al*.^7^ and Keller *et al*.^40^. To date, the most satisfactory performance of ASQ was in Huang *et al*. (AUROC was 0.84, 0.86, and 0.83 for ≥F2, ≥F3, and ≥F4, respectively)^5^. By contrast, the AUROC obtained using ultrasound Nakagami imaging in our study was 0.88, 0.84, 0.69, and 0.67 for ≥F1, ≥F2, ≥F3, and ≥F4, respectively. Ultrasound Nakagami imaging not only improves the diagnostic performance but also efficiently discriminates between the normal and fibrotic stages of the liver tissue, implying that Nakagami imaging has great potential in the early detection of liver fibrosis (≥F1). This finding has not been revealed in previous ASQ literatures.

This study has some limitations that should be addressed in future work. First, the patients were recruited from two hospitals. Therefore, ultrasound scanning and fibrosis scoring were performed by different gastroenterologists and pathologists, respectively. Scoring and scanning errors between data may occur. Second, few patients with histologically proven F0 were recruited. More patients with different types of liver diseases should also be enrolled. Moreover, Nakagami imaging is a model-based ASQ technique for quantifying echo amplitude distribution. Thus, the ASQ-related problems may be encountered when using Nakagami imaging for assessing liver fibrosis. For instance, the lack of a standardized analysis protocol may yield inconsistent results^9^. A coexisting hepatic steatosis may lead to interferences affecting fibrosis detection^39^,^40^. Future studies focusing on establishing a standardized analysis procedure, exploring the effect of steatosis, and validating the reproducibility of ultrasound Nakagami imaging are warranted.

Finally, according to current results and discussion, advantages and limitations of the Nakagami model-based ASQ and the frequently used imaging techniques for liver fibrosis assessment are compared in Table 5. The superiorities of Nakagami imaging over the other methods may include (i) the ability in early detection of liver fibrosis, (ii) using a conventional B-mode machine only as a system requirement, and (iii) potentially a less dependency of inflammation activity because the echo amplitude distribution has no significant correlation with blood alanine aminotransferase level^5^.

### Concluding remarks

Ultrasound Nakagami imaging is a model-based ASQ technique for assessing liver fibrosis. The Nakagami model-based ASQ visualizes changes in the echo amplitude distribution and may have an impact on clinical detection of early liver fibrosis by using standard B-mode ultrasound examinations. We suggest that Nakagami imaging is a complementary tool and may be further combined with elastography in the future. The Nakagami-based ASQ detects early liver fibrosis by imaging the signal amplitude distribution for evaluations of microstructures. Ultrasound elastography is responsible for identifying advanced fibrosis by measuring the liver stiffness. Two-dimensional analysis based on microstructure analysis and stiffness measurement may be a good strategy for future ultrasound diagnosis of liver fibrosis.

## Additional Information

**How to cite this article**: Tsui, P.-H. *et al*. Acoustic structure quantification by using ultrasound Nakagami imaging for assessing liver fibrosis. *Sci. Rep.* **6**, 33075; doi: 10.1038/srep33075 (2016).

## Figures and Tables

**Table 1:** The characteristics of healthy volunteers in the control group.

Characteristics	Value
Male/Female	16/3 (*n* = 19)
Age, years	
Mean ± standard deviation (range)	22.1 ± 1.4 (20–26)
Median	22
BMI, kg/m^2^	
Mean ± standard deviation (range)	23.0 ± 4.4 (17.9–32.9)
Median	20.7

Note—Unless otherwise noted, data are numbers of patients. BMI: body mass index. BMI was calculated and defined according the Department of Health in Taiwan: optimal BMI was defined as 18.5 ≦ BMI < 24 kg/m^2^, overweight as 24 ≦ BMI < 27 kg/m^2^, and obesity was defined as BMI ≧ 27 kg/m^2^.

**Table 2:** Patients’ demographic data and biological and histological findings obtained by liver biopsy examination.

Characteristics	Value
Male/Female	72/19
Age, years	
Mean ± standard deviation (range)	55.4 ± 11.0 (26–75)
Median	58
BMI, kg/m^2^	
Mean ± standard deviation (range)	25.1 ± 5.2 (18.3–65.7)
Median	24.2
AST level, ×ULN	
Mean ± standard deviation (range)	1.4 ± 1.6 (0.4–8.6)
Median	0.8
PLT, 10^3^/mm^3^	
Mean ± standard deviation (range)	187.5 ± 67.9 (70–431)
Median	176
APRI	
Mean ± standard deviation (range)	0.8 ± 0.5 (0.1–2.4)
Median	0.6
HCC	43
HBV infection	40
HCV infection	8
Histological fibrosis stage	
F0	4
F1	19
F2	23
F3	12
F4	33

Note—Unless otherwise noted, data are numbers of patients. BMI: body mass index, AST: aspartate aminotransferase, PLT: platelet count, APRI: AST/PLT, HCC: hepatocellular carcinoma, HBV: hepatitis B virus, HCV: hepatitis C virus, ULN: upper limit of normal. Normal AST levels for female and male subjects are less than 35 U/L and less than 50 U/L, respectively.

**Table 3:** Clinical performance of ultrasound Nakagami imaging in the assessment of liver fibrosis in this study.

Parameter	≧F1	≧F2	≧F3	≧F4
Cutoff value	0.7168	0.7164	0.7025	0.7025
Sensitivity, %	75.86 (65.50 to 84.40)	85.29 (74.61 to 92.72)	71.11 (55.69 to 83.63)	72.73 (54.48 to 86.70)
Specificity, %	91.30 (71.96 to 98.93)	76.19 (60.55 to 87.95)	64.62 (51.77 to 76.08)	59.74 (47.94 to 70.77)
LR+	8.7195	3.5821	2.0099	1.8065
LR−	0.2644	0.1931	0.4471	0.4565
PPV, %	97.10	85.29	58.92	44.64
NPV, %	51.21	76.19	77.77	85.18
AUROC	0.88 (0.79–0.95)	0.84 (0.75–0.92)	0.69 (0.58–0.79)	0.67 (0.56–0.77)

LR+: positive likelihood ratio, LR−: negative likelihood ratio, PPV: positive predictive value, NPV: negative predictive value, AUROC: area under the receiver operating characteristics curve.

**Table 4:** Performances of ASQ in liver fibrosis assessment reported in previous literatures.

Authors	Year	Patients (*n*)	Diseases	AUROC or correlation with fibrosis stages				
				≧F1	≧F2	≧F3	≧F4	Probability value
Toyoda *et al*.^11^	2009	148	HCV	—	—	—	—	<0.05
Ricci *et al*.^39^	2013	77	HBV, HCV	0.71	—	—	0.77	<0.05
Kramer *et al*.^7^	2014	80	HBV, HCV	—	0.46	—	0.38	>0.05
Huang *et al*.^5^	2015	114	HBV	—	0.84	0.86	0.83	<0.001
Huang *et al*.^9^	2015	113	—	—	—	—	—	<0.001
Keller *et al*.^40^	2015	51	HBV, HCV	—	—	—	—	>0.05

AUROC: area under the ROC curve, HCV: hepatitis C virus, HBV: hepatitis B virus.

**Table 5:** A comparison of noninvasive imaging methods in liver fibrosis assessment.

	Advantages or potentials	Limitations
Nakagami model-based ASQ	Superior for detecting early liver fibrosis	May be affected by fatty liver
	A conventional B-mode system is needed only	Worse performance in cirrhosisdetection
	May be less affected by the effect of inflammation	
Ultrasound elastography	Superior for detecting liver cirrhosis	Influence of inflammation
	Good reproducibility	
Computer tomography (CT)	Superior for detecting liver cirrhosis	Radiation dose
		Hard for routine uses
Magnetic resonance (MR) elastography	Superior for detecting fibrosis	Expensive
	No inter-rater variability	Hard for routine uses

Note—Comparisons of Nakagami imaging and ultrasound elastography in the assessment of liver fibrosis are based on the clinical results and discussion in this work. The advantages and limitations of CT and MR elastography are referred to a review paper^41^.
